# Ancestry of the major long-range regulatory site of the α-globin genes in the Portuguese population with the common 3.7 kb α-thalassemia deletion

**DOI:** 10.1007/s11033-024-09530-5

**Published:** 2024-05-05

**Authors:** Rita Pena, Pedro Lopes, Gisela Gaspar, Armandina Miranda, Paula Faustino

**Affiliations:** 1 Departamento de Genética Humana, Instituto Nacional de Saúde Doutor Ricardo Jorge, Avenida Padre Cruz, Lisboa, 1649-016 Portugal; 2 Departamento de Promoção da Saúde e Prevenção de Doenças Não Transmissíveis, Instituto Nacional de Saúde Doutor Ricardo Jorge, Lisboa, Portugal; 3 Grupo Ecogenética e Saúde Humana, Faculdade de Medicina, Instituto de Saúde Ambiental, Universidade de Lisboa, Lisboa, Portugal; 4 Laboratório Associado TERRA, Faculdade de Medicina, Universidade de Lisboa, Lisboa, Portugal

**Keywords:** HS-40 haplotypes, α-Major Regulatory element, Alpha-thalassemia, 3.7 kb deletion

## Abstract

**Background:**

The α-Major Regulatory Element (α-MRE), also known as HS-40, is located upstream of the α-globin gene cluster and has a crucial role in the long-range regulation of the α-globin gene expression. This enhancer is polymorphic and several haplotypes were identified in different populations, with haplotype D almost exclusively found in African populations. The purpose of this research was to identify the HS-40 haplotype associated with the 3.7 kb α-thalassemia deletion (-α3.7del) in the Portuguese population, and determine its ancestry and influence on patients’ hematological phenotype.

**Methods and results:**

We selected 111 Portuguese individuals previously analyzed by Gap-PCR to detect the presence of the -α3.7del: 50 without the -α3.7del, 34 heterozygous and 27 homozygous for the -α3.7del. The HS-40 region was amplified by PCR followed by Sanger sequencing. Four HS-40 haplotypes were found (A to D). The distribution of HS-40 haplotypes and genotypes are significantly different between individuals with and without the -α3.7del, being haplotype D and genotype AD the most prevalent in patients with this deletion in homozygosity. Furthermore, multiple correspondence analysis revealed that individuals without the -α3.7del are grouped with other European populations, while samples with the -α3.7del are separated from these and found more closely related to the African population.

**Conclusion:**

This study revealed for the first time an association of the HS-40 haplotype D with the -α3.7del in the Portuguese population, and its likely African ancestry. These results may have clinical importance as in vitro analysis of haplotype D showed a decrease in its enhancer activity on α-globin gene.

## Introduction

Human hemoglobin is a globular tetrameric protein composed of two α-like and two β-like globin chains. These chains are encoded by two independent gene clusters located in different chromosomal loci: the α-globin gene cluster on chromosome 16 (16p13.3) and the β-globin gene cluster on chromosome 11 (11p15.5). The globin genes in each clusters are organized in the 5’ to 3’ direction, in the same order in which they will be expressed during the different stages of development: embryonic, fetal, and adult [1–3]. The α-globin gene cluster is composed by the embryonic ζ gene (*HBZ*); pseudogenes ψξ (*HBZps*) and ψα1 (*HBA1ps*); two fetal/adult α genes (*HBA2* and *HBA1*); and pseudogene θ (*HBQ*) of unknown function [4, 5].

The high levels and correct expression of the α-globin genes depends on both local and remote *cis*-acting sequences, such as the gene promoter sequences and the α-Upstream Regulatory Element (α-URE), respectively. The α-URE is composed by four highly conserved noncoding regulatory sequences called Multispecies Conserved Sequences (MCS-R1 to MCS-R4) [3, 6, 7]. The major sequence, MCS-R2, also known as HS-40 or α-MRE (α-Major Regulatory Element), is a 350 bp enhancer located 40 kb upstream of the ζ-globin gene Cap site [3, 6], and its main function is to activate and enhance the erythroid lineage-specific and development stage-specific expression of the α-globin genes in *cis* [3, 8–11].

The functional domain of this element is composed by several conserved nuclear binding sites, including two binding sites for the Nuclear Factor Erythroid 2 (NF-E2), three binding sites for GATA-1, and one CACC box, all of which are occupied in vivo in erythroid cells [6, 12]. These regions recruit general transcription factors, as well as the RNA Polymerase II, which binds to the promoter sequence of the α-globin genes [6]. The HS-40 sequence analysis performed by genomic footprinting has demonstrated the formation in vivo of specific nuclear factor DNA complexes at a subset of these sequence motifs in erythroid cells [12]. These transcription factor binding sites showed high conservation between human and other mammals, indicating their functional relevance [13, 14]. However, sequence heterogeneity within or in between these motifs of the human HS-40 fragment occurs between different human populations. Six polymorphic sites in human HS-40 sequence allowed to reconstruct six different combinations designed haplotypes, called A to F. Only A and B haplotypes are present in all groups analyzed. The other haplotypes are present in low frequencies and in specific populations [15, 16]. Haplotype D was primary described in African populations and is nearly absent in other populations [15, 16].

Alpha-thalassemia is an autosomal recessive disorder usually caused by the deletion of one or more α-globin gene that result in a deficiency or absence of α-globin chain synthesis. Alpha-thalassemia is characterized by a microcytic hypochromic anemia, and a clinical phenotype varying from almost asymptomatic to a lethal hemolytic anemia [17]. It is probably the most common monogenic gene disorder in the world and is especially frequent in Mediterranean countries, South-East Asia, Africa, the Middle East and in the Indian subcontinent [17]. Compound heterozygotes and some homozygotes have a moderate to severe form of α-thalassemia called HbH disease. Hb Bart’s hydrops foetalis is a lethal form in which no α-globin chain is synthesized [17].

In the African and European populations, the most common form of α-thalassemia is due to the 3.7 kb deletion, which affects both α-globin genes (*HBA2* and *HBA1*), resulting in a single hybrid gene [17], and the same can be said for the Portuguese population. A study conducted in 1996 using blood samples from 100 newborns showed that 7% of the individuals was heterozygous for the 3.7 kb deletion [18]. On the other hand, large deletions may occur removing all the globin distal regulatory elements as well as the complete α-globin gene cluster, giving rise to total absence of gene expression [17]. Other deletions were described removing only the distal regulatory elements, consequently the *in cis* α-globin genes are physically intact but functionally inactive [19–22]. Very rarely, the deletion that gives rise to α-thalassemia only affects one distal regulatory region, such as the HS-40, leaving the α-globin genes intact but partially inactivated [17, 23–27]. Some of these rare types of deletions that affect the regulatory elements, have also been found in Portuguese individuals, namely the (αα)^MM^, (αα)^ALT^, (αα)^TI^ and (αα)^CSC^ [22, 23, 28].

The HS-40 haplotypes can be used as markers for linkage analyses in addition to common molecular lesions, such as the common 3.7 kb α-thalassemia deletion. Therefore, the main purpose of this study was to characterize the haplotypes of the distal regulatory region HS-40 in individuals with and without α-thalassemia, and identify which haplotype is associated with the 3.7 kb α-thalassemia deletion in the Portuguese population, as well as determine the ancestry of this deletion in this population. Moreover, we intended to investigate if different HS-40 haplotypes are able to affect the hematological phenotype of α-thalassemia due to the homozygosity for the 3.7 kb deletion.

## Materials and methods

### Sample selection

We selected 111 anonymized DNA samples from Portuguese individuals who had already been investigated for the presence of the 3.7 kb α-thalassemia deletion by Gap-PCR as described elsewhere [29]. The criteria for sample selection was based on individuals’ α-globin genotype: wild type, heterozygous for the 3.7 kb deletion, and homozygous for the 3.7 kb deletion. The hematological phenotype of each individual had previously been characterized by standard procedures and included the following hematological parameters: red blood cell count, hemoglobin (Hb) level, mean corpuscular volume (MCV), mean corpuscular hemoglobin (MCH), mean corpuscular hemoglobin concentration, hematocrit, and red cell distribution width. Of the selected individuals, 52 presented with normal hematological parameters, while 59 presented with microcytosis and/or hypochromia.

### DNA extraction

Genomic DNAs were isolated from peripheral blood samples, collected in EDTA, using a nucleic acid automatic extractor, *MagNA pure LC 2.0* (Roche®, Germany). DNA quantity and quality were assessed using a *NanoDrop One* (*Thermo Fisher Scientific*, USA) spectrophotometer. DNAs were stored at 4ºC.

### HS-40 genotyping

To determine the sequence of the different HS-40 haplotypes, a DNA fragment of 400 bp containing the HS-40 region was amplified through conventional PCR, using primers described elsewhere [15]. The amplified PCR fragments were purified using *JET quick PCR Product Purification Spin Kit* (*GENOMED*) according to the manufacturer’s instructions. Sanger sequencing was performed using the *ABI Prism BigDye© Terminator v1.1 Cycle Sequencing* commercial kit (*Applied Biosystems*) in an automated sequencer *3500 Genetic Analyzer* (*Applied Biosystems*). Sequences were analyzed using the *FinchTV v1.4.0* (*Geospiza*) software.

### HS-40 haplotype reconstruction

Six single nucleotide polymorphic sites within the HS-40 fragment characterize the haplotypes A to F in humans. In order to identify the HS-40 haplotypes, the sequence variability observed in our 111 HS-40 fragments was compared to those described by Harteveld and his collaborators in 2002 [15].

### Statistical analysis

The distribution of HS-40 haplotypes and genotypes between the groups of samples was tested using the Test of Equal and Given Proportions. In order to determine the ancestry of the 3.7 kb deletion in the Portuguese population, a multiple correspondence analysis was performed and a specific function to draw the respective graphical representation was used.

For the comparison of the hematological parameters of the individuals with the HS-40 genotypes AA versus AD or DD (using the dominant genetic test model), we started by testing the normality distribution using Shapiro-Wilk’s test. When the normality of both populations was confirmed, the parametric T-test was used. The non-parametric test of Mann-Whitney was applied when there was a non-normal distribution.

All the statistical analysis were performed using *R* software and the statistical significance was established for a *p*-value lower than 0.05.

## Results

### Sample grouping according to the α-globin genotype

The selected 111 Portuguese non-related individuals were divided in three different groups according to their α-globin genotype: 50 without the 3.7 kb α-thalassemia deletion (genotype αα/αα; group 1), 34 with the 3.7 kb deletion in heterozygosity (genotype -α^3.7^/αα; group 2), and 27 with the 3.7 kb deletion in homozygosity (genotype -α^3.7^/-α^3.7^; group 3). All individuals of group 1 present normal levels of Hb, MCV, and MCH. Individuals of group 2 have a mean MCV of 82.7 ± 3.7 fL, MCH of 26.7 ± 2.1 pg, and Hb of 14.8 ± 1.2 g/dL for men and 12.8 ± 0.8 g/dL for women. When it comes to group 3, the individuals present with a mean MCV of 70.4 ± 4.2 fL, MCH of 21.9 ± 1.5 pg, and Hb of 13.9 ± 0.9 g/dL and 11.0 ± 1.0 g/dL for males and females, respectively. Therefore, in the latter group all individuals have hypochromia and microcytosis.

The corresponding DNAs were used to amplify the HS-40 region followed by Sanger sequencing analysis.

### HS-40 genetic findings

The sequence of HS-40 regulatory region in our 111 samples revealed four distinct haplotypes labelled A, B, C, and D (Table 1). Haplotype A (CGCGGG) was the most common in all studied groups (Table 2), which was expected given that this is the ancestral sequence [6, 15]. In general, haplotype B (C**A**C**AG**G) was the second most frequent; being that this was also the second most prevalent haplotype in individuals without α-thalassemia and in the carriers of the 3.7 kb deletion. The very rare haplotype C (C**A**C**AA**G) was only found in three individuals with the wild type α-globin genotype (group 1). On the other hand, haplotype D (CG**T**GGG) was found in nineteen alleles, 78.9% of them from individuals with the 3.7 kb deletion in homozygosity (group 3).

**Table 1:** Polymorphic substitutions in the HS-40 sequence and the corresponding haplotypes found in our samples

HS-40 haplotype	Position of polymorphic sites in the HS-40 sequence					
	+ 96	+ 130	+ 158	+ 199	+ 209	+ 212
***A***	C	G	C	G	G	G
***B***	C	**A**	C	**A**	**G**	G
***C***	C	**A**	C	**A**	**A**	G
***D***	C	G	**T**	G	G	G

Numbers indicate the position of polymorphic sites in the HS-40 sequence, as described [15]

**Table 2:** HS-40 haplotypes in the Portuguese population without the 3.7 kb α-thalassemia deletion (αα/αα), with the deletion in heterozygosity (-α^3.7^/αα) and in homozygosity (-α^3.7^/-α^3.7^)

Alpha-globin genotype	HS-40 haplotypes			
	A x (%)	B x (%)	C x (%)	D x (%)
**αα/αα** (group 1; x = 100)	57 (57.0)	39 (39.0)	3 (3.0)	1 (1.0)
**-α** ^**3.7**^ **/αα** (group 2; x = 68)	46 (67.7)	19 (27.9)	0 (0.0)	3 (4.4)
**-α** ^**3.7**^ **/-α** ^**3.7**^ (group 3; x = 54)	31 (57.4)	8 (14.8)	0 (0.0)	15 (27.8)
Total (x = 222)	134 (60.4)	66 (29.7)	3 (1.3)	19 (8.6)

x = number of alleles

When it comes to the HS-40 genotypes, seven different combinations were found designated AA, AB, AD, BB, BC, BD, and DD (Table 3). We found 53 individuals (57.8%) homozygous for the HS-40 genotype. As they presented the expected hematological phenotype according to their α-globin genotype group, there was no evidence that any of them could be hemizygous rather than homozygous.

The AA and AB combinations were the most common in individuals without α-thalassemia and in those with the -α^3.7^/αα genotype (group 1 and 2), while in patients with the -α^3.7^/-α^3.7^ genotype (group 3) the most prevalent combination was AD.

Haplotype D was found in three different genotypes: AD, BD, and DD. Genotype AD was the most prevalent, being found in 14 individuals, with 71.4% of them also having the 3.7 kb deletion in homozygosity (group 3). In addition, this group is the only one where we can find the very rare genotypes BD and DD.

The distribution of the diverse HS-40 haplotypes and genotypes is significantly different between individuals without α-thalassemia and individuals with the 3.7 kb deletion in homozygosity (*p*-value < 0.001).

**Table 3:** HS-40 genotypes in the Portuguese population without the 3.7 kb α-thalassemia deletion (αα/αα), with the deletion in heterozygosity (-α^3.7^/αα) and in homozygosity (-α^3.7^/-α^3.7^)

Alpha-globin genotype	HS-40 genotypes						
	AA n (%)	AB n (%)	AD n (%)	BB n (%)	BC n (%)	BD n (%)	DD n (%)
**αα/αα** (group 1; *n* = 50)	18 (36.0)	20 (40.0)	1 (2.0)	8 (16.0)	3 (6.0)	0 (0.0)	0 (0.0)
**-α** ^**3.7**^ **/αα** (group 2; *n* = 34)	15 (44.1)	13 (38.2)	3 (8.8)	3 (8.8)	0 (0.0)	0 (0.0)	0 (0.0)
**-α** ^**3.7**^ **/-α** ^**3.7**^ (group 3; *n* = 27)	7 (25.9)	7 (25.9)	10 (37.0)	0 (0.0)	0 (0.0)	1 (3.7)	2 (7.4)
Total (*n* = 111)	40 (36.0)	40 (36.0)	14 (12.6)	11 (10.0)	3 (2.7)	1 (0.9)	2 (1.8)

n = number of individuals

### HS-40 genotype association study with α-thalassemia hematological parameters

In order to investigate if the HS-40 AA, AD, and DD genotypes are influencing the hematological phenotype of individuals with the 3.7 kb α-thalassemia deletion in homozygosity, a statistical comparison between their hematological parameters was performed using the dominant genetic test model (Table 4). However, no significant differences were found (*p*-value > 0.05) for any hematological parameters.

**Table 4:** Statistical comparison between the hematological parameters of Portuguese individuals with the HS-40 genotypes AA *versus* AD or DD, from the group with the 3.7 kb deletion in homozygosity

Hematological Parameters	Group 3 (-α^3.7^/α^3.7^)		p-value
	HS-40 Genotype		
	***AA*** (*n* = 7)	***AD or DD*** (*n* = 12)	
**RBCs (x 10** ^**12**^ **/L)**			
Female Male	5.28 ± 0.24 6.31 ± 0.07	5.62 ± 0.61 4.97	0.514 NA
**Hb (g/dL)**			
Female Male	11.1 ± 0.6 14.1 ± 0.3	10.6 ± 0.6 11.8	0.647 NA
**HCT (%)**	36.7 ± 2.3	36.8 ± 1.4	0.501
**MCV (fL)**	69.0 ± 2.4	72.1 ± 0.8	0.264
**MCH (pg)**	21.7 ± 0.9	22.2 ± 0.2	0.855
**MCHC (g/dL)**	31.4 ± 0.4	20.6 ± 0.3	0.139
**RDW (%)**	17.1 ± 1.1	14.9 ± 0.4	0.105

n = number of individuals; NA – Not Applicable (only one male individual with genotype AD or DD, not enough observations); RBC – Reb Blood Cells; Hb – Hemoglobin; HCT – Hematocrit; MCV – Mean Corpuscular Volume; MCH – Mean Corpuscular Hemoglobin; MCHC – Mean Corpuscular Hemoglobin Concentration; RDW – Red Cell Distribution Width

### Ancestry of the 3.7 kb α-thalassemia deletion in the Portuguese population

After determining that the specific HS-40 haplotype D and genotypes AD, BD, and DD, are associated with the presence of the 3.7 kb α-thalassemia deletion in the Portuguese population, we aimed to investigate the ancestry of this deletion in this population. Initially, these genotypes were only reported in African people [15, 16], however more recently, they were also detected in Uruguayans [30]. In the two populations, these genotypes have been found mostly in individuals with the 3.7 kb deletion.

**Fig. 1:**
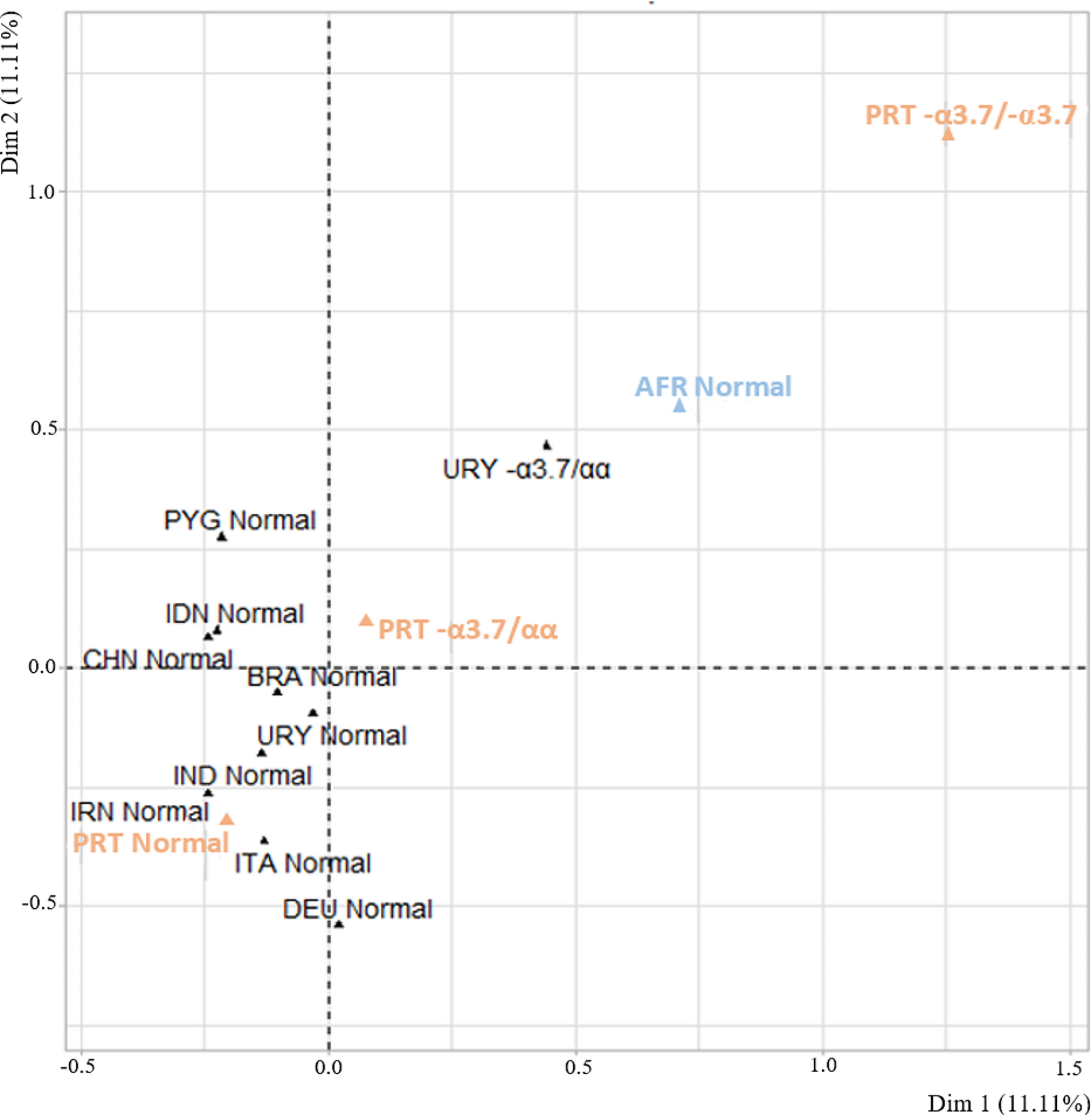
Multiple correspondence analysis of the -α^3.7^ genotypes in multiple geographic populations. AFR: African; BRA: Brazilian; CHN: Chinese; DEU: Dutch; IDN: Indonesian; IND: Indian; IRN: Iranian; ITA: Italian; PRT: Portuguese; PYG: Pygmies; URY: Uruguayan. All the genotypes from foreign populations were collected from [15, 16, 30, 31]. The Portuguese populations investigated in this study are marked as PRT normal, PRT -α^3.7^/αα, and PRT -α^3.7^/-α^3.7^

Multiple correspondence analysis was performed in order to better visualize the similarities between the Portuguese population and other populations [15, 16, 30, 31]. This analysis showed that the Portuguese individuals who do not have α-thalassemia (PRT Normal) are grouped with other European populations, while samples with the 3.7 kb deletion (PRT -α3.7/αα and PRT -α3.7/-α3.7) are isolated from these and found to be more closely related to the African population (Fig. 1).

## Discussion

The Portuguese population is predominantly composed of haplotype A (60%) and haplotype B (30%), according to the 222 alleles analyzed for the HS-40 region sequence. Similarly, haplotype A was also reported as predominant in the Italian, Indonesian, Chinese, East Indian, Bantu-speaking-Africans, Brazilian Indians, and Uruguayan populations, with frequencies ranging from 56 to 87% [15, 16, 30], while haplotype B was found to be predominant exclusively in the Dutch population (57%) [15]. For the other populations indicated above, the haplotype B frequencies are lower and range between 13 and 43% [15, 16, 30]. On the other hand, haplotype D is characteristic of Bantu-speaking Africans (16%) and Pygmies from the Central African Republic (5%), being nearly absent in others populations [15]. Nonetheless, a high frequency of haplotype D was found in the Uruguayan population (6.4%) [30] and here in this study (8.6%). Furthermore, our results showed that the distribution of HS-40 haplotypes and genotypes are significantly different between individuals with and without the 3.7 kb α-thalassemia deletion and, consequently, that there is an association between the HS-40 haplotype D and the presence of this deletion in the Portuguese population. For this conclusion, it certainly weighs a lot the presence of haplotype D, as well as the genotypes AD, BD, and DD, that were found mainly in individuals with the -α^3.7^/-α^3.7^ genotype. Thus, we hypothesize that the significant higher frequency of haplotype D in the sample with the -α^3.7^ deletion may be due to a predominant African origin of this deletion in the Portuguese population. The same was concluded for the Uruguayan population [30].

Haplotype D derived from haplotype A by a nucleotide substitution at position + 158, which leads to a change in the consensus sequence for the AP-1/NF-E2 binding site, a composite binding site that is recognized by the transcription factor NF-E2 [32, 33]. Previous studies using murine erythroleukemia cells revealed that this transcription factor acts as an enhancer-binding protein for long-range regulation of globin gene expression and that, consequently, α-globin gene expression is highly dependent on NF-E2 [34–36]. Besides that, analysis of mice lacking NF-E2 showed that these mice exhibit some microcytosis, increase erythropoiesis, mild anemia, and their red cells present a slight decrease in hemoglobin content [34, 37]. Furthermore, other studies showed that mutated AP-1/NF-E2 binding sites lead to a 25% reduction in α-globin gene expression in transgenic mice [38], and in vitro experiments using constructs with the luciferase gene under the control of the different human HS-40 haplotypes revealed a noticeable reduction in luciferase expression in all haplotypes compared to A haplotype [39].

Consequently, interference in the NF-E2 binding site, as seen in haplotype D, may result in decreased α-globin gene expression in humans; even so, the presence of this HS-40 haplotype in heterozygosity is not enough to cause α-thalassemia. Moreover, the interference with this transcription factor binding site may have a greater impact in individuals that either have the genotype DD or that have a combination of haplotype D and an α-thalassemia defect, such as the 3.7 kb deletion. It was hypothesized that in individuals homozygotes for both the HS-40 haplotype D and the 3.7 kb deletion, α-globin gene expression may reduce below a critical level and result in the formation of HbH (β4 tetramers), due to an excess of unpaired β-globin chains [15]. However, our results did not reveal a significant difference between the hematological parameters of individuals with the HS-40 AA, AD, or DD genotypes, and with homozygosity for the 3.7 kb α-thalassemia deletion. Similar results were obtained by Harteveld and collaborators [15]. These may be justified by many reasons, one of them may be the sample size being too small to draw any conclusions, since patients homozygous for both HS-40 DD and -α^3.7^/-α^3.7^ are rare. Alternatively, the long-range regulation of α-globin gene expression in mice may differ from that in humans, as suggested by other studies [3, 23, 40], and is probably under a more complex mechanism, which may include epigenetic regulations.

Furthermore, in this study, a multiple correspondence analysis revealed that Portuguese individuals without α-thalassemia are grouped with other European populations, while samples with the 3.7 kb deletion are separated from these and more closely related to the African population, which reinforces the previous hypothesis and leads to the conclusion that there is a predominant African origin of the 3.7 kb α-thalassemia deletion in the Portuguese population.

## Conclusion

In conclusion, this study revealed for the first time an association between the HS-40 haplotype D and the common 3.7 kb α-thalassemia deletion in the Portuguese population, and its likely African ancestry. This result contribute to the knowledge of the different genetic background between populations. Furthermore, this work highlights the importance of further studies to know better the consequences of genetic variability on the long-range regulation of α-globin genes in humans. The related experiments, carried out in vitro or in transgenic mice, revealed results that suggest clinical consequences, but these have not yet been validated in humans.
